# Implementation of new concise care pathways into a software tool to detect and manage comorbidities in older patients with atrial fibrillation: the Horizon 2020 EHRA-PATHS project

**DOI:** 10.1093/europace/euaf111

**Published:** 2025-10-27

**Authors:** Rana Önder, Lien Desteghe, Eline Vandeput, Michelle Lobeek, Michiel Rienstra, Johan Vijgen, Hein Heidbuchel, for the EHRA-PATHS investigators, Lars Grieten, Eline Vandeput, Inge Liebens, Filip Aben, Giuseppe Boriani, Serge Boveda, Ronan Collins, Rafal Dabrowski, David Duncker, Stavros Karanikas, Ester Maas-Soer, Katarzyna Malaczynska-Rajpold, Sime Manola, José Merino, Bart Mulder, Mario Oliveira, Borka Pezo, Helmut Pürerfellner, Michiel Rienstra, Maciej Sterlinski, Milko Stoyanov, Emma Svennberg, Vassil Traykov, Stelios Tzeis, Colinda van Deutekom, Isabelle van Gelder

**Affiliations:** Faculty of Medicine and Life Sciences, Hasselt University, Martelarenlaan 42 Hasselt 3500, Belgium; Heart Centre Hasselt, Jessa Hospital, Stadsomvaart 11 Hasselt 3500, Belgium; Faculty of Medicine and Life Sciences, Hasselt University, Martelarenlaan 42 Hasselt 3500, Belgium; Heart Centre Hasselt, Jessa Hospital, Stadsomvaart 11 Hasselt 3500, Belgium; Department of Cardiology, Antwerp University Hospital, Drie Eikenstraat 655 Edegem 2650, Belgium; Centre for Research and Innovation in Care (CRIC), Department of Nursing and Midwifery Sciences, University of Antwerp, Prinsstraat 13 Antwerp 2000, Belgium; Research Group Cardiovascular Diseases, GENCOR, University of Antwerp, Prinsstraat 13 Antwerp 2000, Belgium; Qompium NV, Kempische Steenweg 303/27 Hasselt 3500, Belgium; Department of Cardiology, University of Groningen, University Medical Centre Groningen, Hanzeplein 1 GZ 9713, Groningen, The Netherlands; Department of Cardiology, University of Groningen, University Medical Centre Groningen, Hanzeplein 1 GZ 9713, Groningen, The Netherlands; Faculty of Medicine and Life Sciences, Hasselt University, Martelarenlaan 42 Hasselt 3500, Belgium; Heart Centre Hasselt, Jessa Hospital, Stadsomvaart 11 Hasselt 3500, Belgium; Faculty of Medicine and Life Sciences, Hasselt University, Martelarenlaan 42 Hasselt 3500, Belgium; Department of Cardiology, Antwerp University Hospital, Drie Eikenstraat 655 Edegem 2650, Belgium; Research Group Cardiovascular Diseases, GENCOR, University of Antwerp, Prinsstraat 13 Antwerp 2000, Belgium

**Keywords:** Atrial fibrillation, Multimorbidity, Software, Care pathways

**This Rapid Communication is linked to the Original Article ‘Development of three-step holistic care pathways to detect and manage comorbidities in patients with atrial fibrillation: the Horizon 2020 EHRA-PATHS consortium' by R Önder**  ***et al.*****, https://doi.org/10.1093/ehjopen/oeaf120 and the Editorial ‘The complexity of tackling multimorbidity in Atrial Fibrillation How European projects are reshaping our approach to comorbidities’ by G Lip**  ***et al.*****, https://doi.org/10.1093/ehjopen/oeaf132 published in**  ***European Heart Journal Open***.

## Introduction

Atrial fibrillation (AF) is often caused by and/or associated with underlying comorbidities that affect its progression and outcomes.^1^ An average of five comorbidities is typical for older patients with AF (≥65 years).^2,3^ In clinical practice, there are no tools to systematically help track and manage comorbidities.^4–6^ The EHRA-PATHS Horizon-2020 project,^7,8^ executed by a consortium of 14 partners from 11 European countries, focuses on developing systematic, structured, and interdisciplinary care pathways to fill this void. In another paper, we reported on the process of developing a consensus on the content of 23 concise care pathways to detect and address multimorbidity in older patients with AF.^9^ Here, we report how these care pathways were implemented in an easy-to-use web-based software tool for daily clinical practice.

## Steps and timeline of software development

**1. Preparatory steps**

The EHRA-PATHS project started on 1 March 2021, after being awarded in fall 2020. Software development (Work Package 4) was initially planned to start 18 months after the start of the project but was launched a year earlier (10/2021) and ran into June 2023. Minor additions and bug fixes were later added, focusing on the electronic case report form (eCRF) functionality desired for the randomized clinical trial (RCT) of Work Package 5.

In the first phase (10/2021 till 3/2022), the Antwerp University Hospital (UZA) team had exploratory meetings with companies to understand the process of software development within a tight timeline and budget. These discussions highlighted the need for a formal discovery phase to define the software scope, requirements, and development priorities.

UZA initiated a discovery cycle, with Qompium (Hasselt, Belgium) hired as a consultant, from March to June 2022, involving meetings between UZA, University Medical Centre Groningen (UMCG), and Qompium. This phase resulted in a comprehensive list of software requirements, including regulatory aspects, functionalities, user groups, and clinical trial needs.

The discovery phase enabled an official European tender procedure conducted by UZA’s Purchasing and Legal Departments from July to September 2022, with four companies participating. Predefined evaluation included previous experience with similar projects, adherence to guidelines (like European Medical Device Regulation, General Data Protection Regulation, and ISO-certification standards), integration with eCRFs across Europe, absence of conflicts of interest, post-development support, compliance with deadlines and budget as foreseen in the EU Grant. Ultimately, Qompium was selected as best fulfilling all requirements, and software development started in October 2022 after the formal assignment.

**2. Development phase**

The EHRA-PATHS software is built on the backbone of Qompium’s CE-marked and medically approved FibriCheck application, which measures heart rate and regularity via photoplethysmography for AF detection.^10^ The existing FibriCheck software functionalities concerning security and privacy, a healthcare providers’ (HCP) dashboard, and patient interaction were customized for EHRA-PATHS (without the heart rhythm functionalities of FibriCheck). The discovery phase requirements were expanded by additional features for the planned RCT (ClinicalTrials.gov NCT05773768), and the 23 comorbidities were divided into two groups based on their assessment as RCT endpoint.^9^ Development had a tight deadline (10/2022-3/2024) necessitating an intensely iterative approach for the clinical project partners: (i) Qompium created prototypes with progressively more functional capabilities spanning more care pathways, which were discussed in bi-weekly meetings with UZA and UMCG teams. (ii) The UZA and Qompium teams performed extensive internal software testing, with UZA focusing on the implementation and logic of the care pathways and reporting bugs to Qompium. (iii) In April and May 2022, the 12 clinical consortium partners tested the prefinal software and provided feedback on the content of the care pathways, logic, spelling errors, and unclear terms, while UZA compiled a software manual. Moreover, the partners completed a seven-question survey rating their satisfaction with the software’s manual, content, logic, and layout, on a scale of 0–10. Finally, assessors were asked to estimate the number of sessions and time that they thought would be required to complete the detection triggers (i.e. the first step in the software). After this phase, bugs and major suggestions were implemented to finalize the software in summer 2023. AF nurses then used the final software to pilot-test the coding of 24 patients in clinical care.

## Results of the survey among the consortium partners

**Figure 1:**
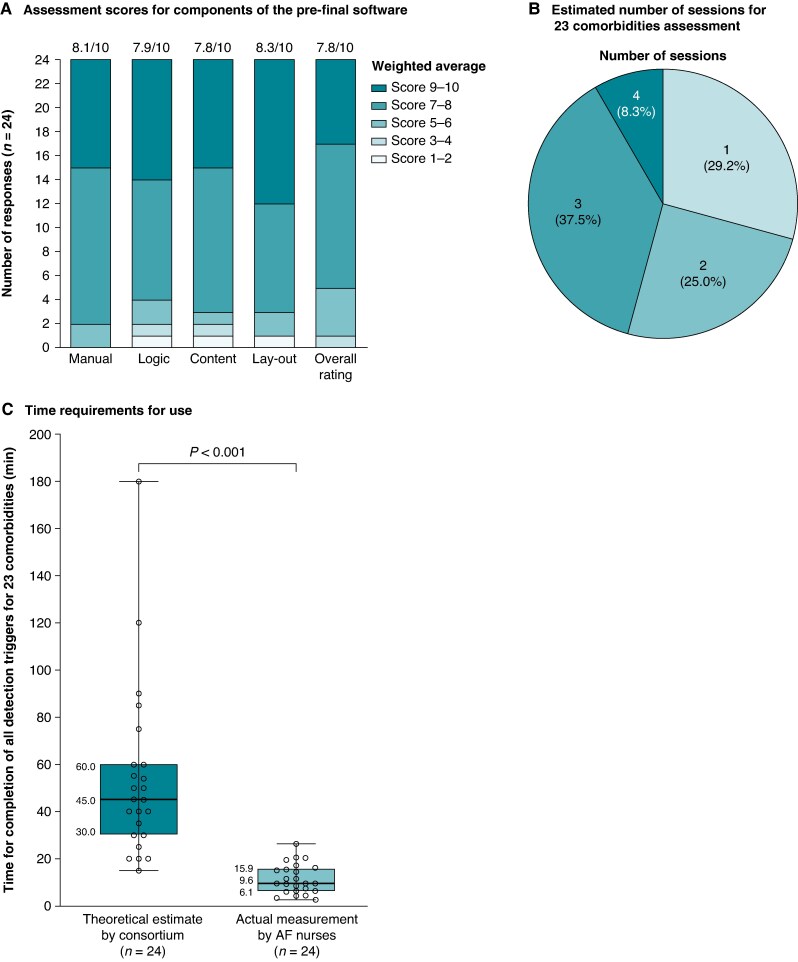
Software evaluation by the consortium based on the survey results and the time needed to complete the detection trigger (*n* = 24): (*A*) Scores for each aspect of the software evaluation by the consortium with the weighted average; (*B*) Estimated number of sessions needed to complete all detection triggers by the consortium; (*C*) Time needed to complete all 23 detection triggers with the median (interquartile range), estimated by the consortium members (*n* = 24), and actual time measured in practice by AF nurses in 24 patients. Mann–Whitney *U* test.

In total, 24 responses from 10 countries were received during the software’s final evaluation (13 cardiologists, 6 nurse specialists, 2 researchers, 1 general practitioner, 1 technician, and 1 clinical pharmacist). HCPs were positive about the software’s content (weighted average, 7.9/10), implemented logic (7.8/10), layout (8.3/10), manual (8.1/10), and overall functionality (7.8/10) (*Figure 1A*). Eleven evaluators (45.8%) reported software errors that were later corrected/addressed. Without testing it in a clinical context, HCP estimated that an average of two sessions would be needed to input all information to check for the presence of 23 comorbidities in a new AF patient (i.e. the first phase) (*Figure 1B*), with an estimated median completion time of 45.0 min. Interestingly, in the ensuing pilot test by AF nurses in clinical practice, the actual completion of the detection triggers took only 9.6 min (*P* < 0.001) (*Figure 1C*).

## Conclusions

Software development for our project, a process unfamiliar to most clinical investigators, faced a number of challenges: an ambitious timeline, a fixed budget, regulatory compliance requirements for future use in Europe, and eCRF functionalities for the RCT. An early start, an essential discovery phase, dedicated clinical research teams, and strict adherence to the timeline and budget contributed to the timely and successful completion of the software development. The assessment of the final product by the consortium was deemed satisfactory, with only minor optimizations needed for the RCT. Fortunately, the time needed to complete the detection trigger is significantly lower than theoretical estimates. Efforts to further optimize this process are ongoing, including finalizing a checklist to anticipate and facilitate software completion. This is essential for further reducing completion time, which is crucial for routine clinical practice. The software is ready for testing in a clinical setting through the EHRA-PATHS RCT, but its acceptance as a medical device varies across EU member states. We are confident that our efforts will lead to a standardized and efficient clinical approach for comorbidity assessment and management of patients with AF.

## Data Availability

No new data were generated or analysed in support of this research.
